# Complete genome sequence of *Streptococcus vaginalis* strain UMB8616 isolated from the bladder of a female with urge urinary incontinence

**DOI:** 10.1128/mra.00661-26

**Published:** 2026-08-11

**Authors:** Brian I. Choi, Helen Appleberry, Melline Fontes Noronha, Catherine Putonti, Alan J. Wolfe

**Affiliations:** 1 Department of Microbiology and Immunology, Stritch School of Medicine, Loyola University Chicago 12248 , Maywood, Illinois, USA; 2 Bioinformatics Program, Loyola University Chicago 2456, Chicago, Illinois, USA; Fluxus Inc., Sunnyvale, California, USA

**Keywords:** *Streptococcus*, *Streptococcus vaginalis*, urge urinary incontinence, human microbiome, urobiome, complete genome

## Abstract

*Streptococcus vaginalis* is a recently identified bacterial species closely related to *Streptococcus anginosus*. It has been isolated from the human urogenital tract. We report the complete genome sequence of *S. vaginalis* UMB8616 (=ATCC TSD-371 = CCUG 77169 = DSM 115471) isolated from the bladder of a human female with urge urinary incontinence.

## ANNOUNCEMENT

*Streptococcus vaginalis* is a gram-positive, non-spore-forming, catalase-negative, facultative anaerobic cocci that was recently identified as a novel species most closely related to *Streptococcus anginosus* (1). *S. vaginalis* distinguishes itself from *S. anginosus* by its ability to ferment D-raffinose and mannitol (1). The species has not been formally recognized as a part of the Anginosus group consisting of *S. anginosus*, *S. constellatus*, and *S. intermedius* (2). However, *S. vaginalis* has been isolated from the same human urogenital niches as other Anginosus group members (1, 3, 4). A previous study analyzing urinary tract-derived *S. anginosus* strains that had the ability to acidify raffinose characterized a unique gliding-type motility phenotype (5). Such studies indicate that strains previously identified as *S. anginosus* may require reanalysis with this new species identification.

In this study, we present a *S. vaginalis* strain UMB8616 (=ATCC TSD-371 = CCUG 77169 = DSM 115471) isolated from a female diagnosed with urge urinary incontinence (UUI) from bladder urine obtained via transurethral catheter from a female urogynecology patient at Loyola University Medical Center, Maywood, Illinois, United States as part of a prospective clinical study (NCT02495389) (IRB#: LU208983) (6). This method involved incubating urine on sheep blood agar plates for 48 h at 37°C with 5% supplemented CO_2_ gas, followed by single-colony isolation. The strain was originally identified as *S. anginosus* via matrix-assisted laser desorption/ionization time-of-flight mass spectrometry (MALDI-TOF), as previously described (7). For isolation of DNA, a sample was cultured from cryopreservation using the same conditions and method as identification.

DNA for short-read Illumina sequences was extracted with the Qiagen UltraClean Microbial Kit according to the manufacturer’s instructions. Sequencing was conducted using the Nextera XT DNA Library Preparation Kit (Illumina) and the MiSeq Reagent Kit v2 run on the Illumina MiSeq platform. DNA extraction and sequencing for long-read Oxford Nanopore sequences was performed by SeqCenter using ZymoBIOMICS DNA Miniprep Kit and Oxford Nanopore Technology Ligation Sequencing Kit V14 with the NEBNext Companion Module. No additional DNA fragmentation or size selection was performed. Sequencing was conducted on R10.4.1 flowcells run on the MinION platform. Guppy v6.4.6 (Nanopore) was used for basecalling, demultiplexing, and adapter removal.

Hybrid assembly was performed by combining short and long sequence reads. Default parameters were used for all software, unless otherwise specified. Raw read quality was assessed using FastQC v0.11.9 (8). Low-quality Illumina and Oxford Nanopore reads were filtered using Cutadapt v3.7 (9) and NanoFilt v2.8.0 (10), respectively. Long reads were used for the initial genome assembly with Flye v2.9 (11). Illumina reads were subsequently used for polishing with Pilon v1.24 (12). Genome assembly quality was evaluated using QUAST v5.2.0 (13), and genome circularization and rotation to *dnaA* were performed with Circlator v1.5.5 (14). Genome completeness and contamination were assessed using CheckM v1.2.0 (15), applying the lineage-specific workflow (lineage_wf). Prokaryotic Genome Annotation Pipeline (PGAP) v6.7 (16) was used for genome annotations. Species was confirmed via digital DNA:DNA hybridization (17); *S. vaginalis* UMB8616 and *S. vaginalis* P1L01^T^ (GCF_017315345.1) have a *d*4 value of 94.7%, which exceeds the species threshold (18).

Table 1 presents the characteristics of the complete *S. vaginalis* UMB8616 chromosome and its plasmid pUMB8616_1. Availability of this complete genome is foundational for subsequent reanalysis of *S. anginosus* genomes.

**TABLE 1:** Complete genome characteristics

Characteristic	*S. vaginalis* UMB8616
Illumina sequencing	
Read length (nucleotides)	250
No. of read pairs	851,883
Nanopore sequencing	
No. of reads	220,575
Read *N*_50_ (bp)	8,170
Annotation statistics	
No. of contigs	2
Structure	1 chromosome (circular), 1 plasmid (circular)
Genome length (bp), chromosome (plasmid)	2,033,097 (9,612)
GC content (%)	39.15
Average coverage (×)	711
Predicted no. of coding DNA sequences	1,938
No. of rRNAs (5S, 16S, 23S)	4, 4, 4
No. of tRNAs	60

## Data Availability

The Illumina raw reads/Nanopore raw reads/assembly have been deposited in GenBank under the following accession numbers: SRR10153371/SRR25068137/GCA_008726365.3. The complete genome of the chromosome sequence is deposited under GenBank accession number CP136160.1 and the complete plasmid sequence, pUMB8616_1, under GenBank accession number CP136161.1.
